# Artificial Intelligence and Hysteroscopy: A Multicentric Study on Automated Classification of Pleomorphic Lesions

**DOI:** 10.3390/cancers17152559

**Published:** 2025-08-03

**Authors:** Miguel Mascarenhas, Carla Peixoto, Ricardo Freire, Joao Cavaco Gomes, Pedro Cardoso, Inês Castro, Miguel Martins, Francisco Mendes, Joana Mota, Maria João Almeida, Fabiana Silva, Luis Gutierres, Bruno Mendes, João Ferreira, Teresa Mascarenhas, Rosa Zulmira

**Affiliations:** 1 Department of Gastroenterology, São João University Hospital, 4200-319 Porto, Portugalmiguelpedro96@gmail.com (M.M.); francisco.cnm@gmail.com (F.M.); joanamfmota8@gmail.com (J.M.);; 2 WGO Gastroenterology and Hepatology Training Center, 4200-319 Porto, Portugal; 3 Faculty of Medicine, University of Porto, 4200-319 Porto, Portugal; 4 Department of Gynecology, São João University Hospital, 4200-319 Porto, Portugal; carlapcp@gmail.com (C.P.); tqc@sapo.pt (T.M.); 5 Department of Gynecology, Ambulatório Médico de Especialidades Barradas, São Paulo 6479200, Brazilluisf.gutierres@gmail.com (L.G.); 6 Department of Gynecology, Centro Materno-Infantil do Norte Dr. Albino Aroso (CMIN), Santo António University Hospital, 4050-651 Porto, Portugalrosazulmira@gmail.com (R.Z.); 7 Department of Mechanical Engineering, Faculty of Engineering, University of Porto, 4200-465 Porto, Portugal; brunomendes81@gmail.com (B.M.); jferreira@fe.up.pt (J.F.)

**Keywords:** endometrial polyps, hysteroscopy, gynecology, artificial intelligence

## Abstract

**Background/Objectives**: The integration of artificial intelligence (AI) in medical imaging is rapidly advancing, yet its application in gynecologic use remains limited. This proof-of-concept study presents the development and validation of a convolutional neural network (CNN) designed to automatically detect and classify endometrial polyps. **Methods**: A multicenter dataset (n = 3) comprising 65 hysteroscopies was used, yielding 33,239 frames and 37,512 annotated objects. Still frames were extracted from full-length videos and annotated for the presence of histologically confirmed polyps. A YOLOv1-based object detection model was used with a 70–20–10 split for training, validation, and testing. Primary performance metrics included recall, precision, and mean average precision at an intersection over union (IoU) ≥ 0.50 (mAP50). Frame-level classification metrics were also computed to evaluate clinical applicability. **Results**: The model achieved a recall of 0.96 and precision of 0.95 for polyp detection, with a mAP50 of 0.98. At the frame level, mean recall was 0.75, precision 0.98, and F1 score 0.82, confirming high detection and classification performance. **Conclusions**: This study presents a CNN trained on multicenter, real-world data that detects and classifies polyps simultaneously with high diagnostic and localization performance, supported by explainable AI features that enhance its clinical integration and technological readiness. Although currently limited to binary classification, this study demonstrates the feasibility and potential of AI to reduce diagnostic subjectivity and inter-observer variability in hysteroscopy. Future work will focus on expanding the model’s capabilities to classify a broader range of endometrial pathologies, enhance generalizability, and validate performance in real-time clinical settings.

## Simple Summary

Hysteroscopy is subject to significant intra- and inter-observer variability due to the wide range of endometrial lesions that can be encountered. The application of artificial intelligence (AI) offers a promising avenue to mitigate this variability; however, its development in gynecology remains in its early stages compared to other medical imaging fields. In this study, we developed an AI model using a multicentric and diverse dataset, which demonstrated high performance not only in detecting polyps but also in accurately classifying them. Moreover, the use of bounding boxes provides visual localization that can potentially be deployed in real-time clinical procedures. Therefore, while AI adoption in gynecology is still emerging, this study illustrates its feasibility and clinical promise.

## 1. Introduction

The use of artificial intelligence (AI) is rapidly transforming various aspects of medicine, particularly in areas that involve image analysis [1]. Gynecology, a field heavily reliant on medical imaging, stands to benefit significantly from these developments [2].

Gynecologic cancers represent roughly 11% of new cancer diagnoses in the United States, and cervical, endometrial, and ovarian cancers rank among the most common [1,3,4,5]. Endometrial cancer is often diagnosed at an early stage but mortality rates have been increasing for the last 25 years [6,7].

When evaluating and managing women with suspected intrauterine pathology, gynecology guidelines recommend the use of hysteroscopic techniques [8,9,10]. Hysteroscopy is a crucial diagnostic procedure in gynecology and consists of inserting a thin, lighted tube with a camera into the uterus to examine the uterine lining for abnormalities. It involves the real-time transmission of images from the hysteroscope to a screen, allowing the gynecologist to guide the instrument and evaluate the cavity. Each procedure lasts about 5 and 30 min, depending on the indication, findings, and the need for therapeutic intervention, and is captured as a continuous video [11,12]. This complete video sequence is frequently retained by healthcare facilities for follow-up, such as comparing diagnoses over time, consulting with other specialists, or contributing to research studies [13]. This procedure is indicated in the evaluation of various conditions, including endometrial polyps, fibroids, abnormal bleeding, and suspected malignancies [11,14,15]. However, traditional visual interpretation of hysteroscopy images poses challenges such as subjectivity and inter-observer variability, potentially hindering accurate diagnosis.

Despite the potential of AI in various imaging-dependent medical areas, its impact on gynecologic imaging remains relatively limited [16,17,18]. With growing interest in this field, a recent paper reviewed the state of the art in AI applications to gynecology, highlighting AI’s potential across various gynecologic specialties, from urogynecology to oncology [19].

Deep learning techniques, such as convolutional neural networks (CNNs), are AI-driven architectures inspired by the human brain’s visual processing. They have emerged as powerful tools with high proficiency for image pattern detection [20,21,22]. Therefore, deep learning algorithms, namely CNNs, are a great promise in the field of gynecology, allowing imaging recognition, reconstruction, processing, automated analysis, and classification. Numerous examples of such systems using endoscopic images in the diagnosis of gastric and colon lesions have been published and commercialized [23]. However, no such system has been developed with specific focus on endometrial pathology [19].

By training CNNs on large datasets of labeled hysteroscopy images, these algorithms can learn to differentiate between normal and abnormal findings. In fact, despite AI application potential in gynecology, data on the use of AI in hysteroscopy procedures are lacking with only a few papers published on the topic [19]. The aim of this proof-of-concept study was to develop a CNN-based algorithm for the automatic detection and classification of polypoid lesions in hysteroscopy images.

## 2. Materials and Methods

### 2.1. Ethical Considerations

This study respected the Declaration of Helsinki and was developed in a non-interventional fashion. The study was approved by the ethical committee (IRB 2023.157 (131-DEFI/123-CE)). Omission of potentially identifying information of the subjects was ensured and each patient received a random number assignment to obtain effective data anonymization for researchers involved in the CNN development.

Given the retrospective and non-interventional nature of this study, informed consent was waived by the review boards of the participating centers. All data were fully anonymized in compliance with institutional policies and general data protection regulation (GDPR) regulations, and the use of diagnostic materials for research purposes without individual consent is allowed under these conditions. A legal team with data protection officer certification was responsible for the non-traceability of the data in conformity with GDPR.

### 2.2. Study Design and Dataset Preparation

This study included hysteroscopies performed at three centers: Unidade Local de Saúde de Santo António (ULSSA) between June 2023 and February 2024, Unidade Local de Saúde de São João (ULSSJ) between January 2024 and April 2024, and at Ambulatório Médico de Especialidades Barradas São Paulo (AME Barradas SP) between January 2024 and December 2024. A total of 65 procedures were used for the development of the CNN (6 from ULSSA, 26 from ULSSJ, and 33 from AME Barrradas). In ULSSA the hysteroscopies were performed with a Storz hysteroscope and EDDY 3D processor, in ULSSJ the hysteroscopies were performed with a Braun hysteroscope and EDDY 3D processor, and in AME Barradas SP the hysteroscopies were performed with a Bettocchi Storz hysteroscope and Confiance Medical CM-SCAM3 processor. All procedures were performed by experienced gynecologists, following clinical standards including biopsy and resection when indicated.

Still frames were extracted from hysteroscopy procedures of ULSSA and AME Barradas SP, while full videos from ULSSJ were segmented into still frames. After a comprehensive review process, we compiled a dataset consisting of 33,239 frames. Only frames with visible histological-confirmed polyps were annotated, resulting in 37,512 object-level annotations across 33,174 frames. A small number of non-polyp frames (n = 65) were included to support model training.

**Figure 1:**
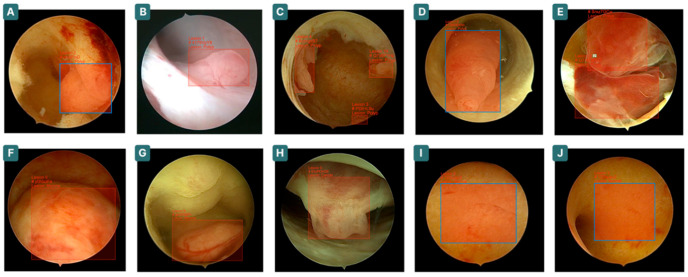
Representative images of lesion detection and classification by the CNN. The images display real outputs with bounding boxes identifying detected areas and classifying findings. Examples include polyps (**A**–**E**), myomas (**F**,**G**), cancer (**H**), and normal endometrium (**I**,**J**).

**Figure 2:**
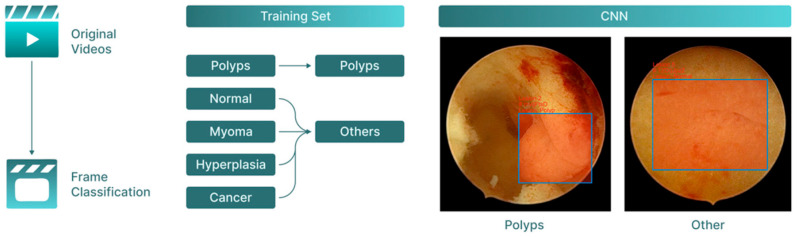
Classification flow chart. From the original hysteroscopy videos, individual frames were extracted and classified into two categories—polyps and ‘other’ findings (including normal endometrium, myomas, and cancer). These annotated frames formed the training dataset used to develop the CNN, which was subsequently applied to classify new, unseen frames.

Due to the limited number of cases available for individual lesion types, a binary classification scheme was adopted: polyp vs other (including normal endometrium, myomas, and cancer). Object-level labels were confirmed through histological reports. All annotations were performed by two experienced physicians (see Figure 1 and Figure 2). Frames with ambiguous findings, typically due to low image quality or blurring, were re-evaluated by both annotators. When consensus could not be achieved, these frames were excluded from the dataset. Although the number of discarded frames was not formally recorded, retrospective estimates indicate that these exclusions affected less than 1% of the total dataset. This approach ensured that only high-confidence annotations were used for model development.

**Figure 3:**
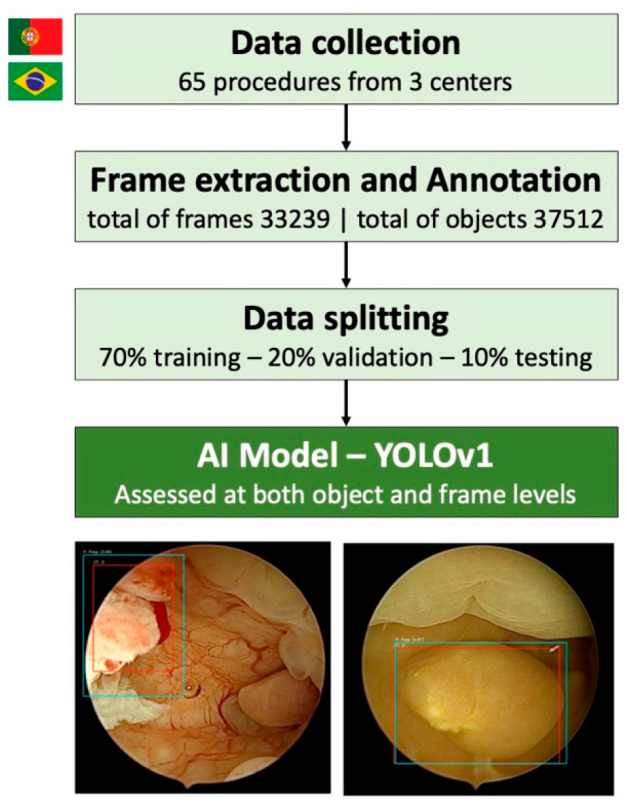
Study design flowchart. Videos were collected from three centers; frames were extracted and annotated for polyps versus other lesions. Data were then split 70% training, 20% validation, 10% test at the object level; finally, a CNN was trained and tested for automated detection and classification.

The dataset was randomly split into three parts—a training set (70%), a validation set (20%), and a testing set (10%). The split was performed at an object level. The validation set was used for model tuning, while the testing set was used for final performance assessment of the model (Figure 3).

### 2.3. Model Development and Evaluation

A YOLOv1-based object detection model was trained to automatically detect and localize polyps in hysteroscopic images. YOLO means ‘you only look once’ and is an AI model that can quickly detect and locate objects in an image in a single step [24]. Unlike traditional methods, it identifies what is in the image and where it is at the same time. The model divides each image into a grid, with each cell predicting bounding boxes and class probabilities simultaneously. This single-stage architecture allows real-time inference and efficient performance. Each image was processed by a CNN, which extracted hierarchical features and mapped onto a grid of fixed-size. Each grid cell was responsible for predicting bounding boxes and class probabilities for objects centered within it. Non-maximum suppression (NMS) was applied to eliminate redundancy or overlapping predictions. The intersection over union (IoU), defined as the overlap between predicted and annotated boxes, is used to evaluate detections. Only predictions with the highest confidence scores and sufficient IoU values are retained. The confidence and IoU thresholds were fine-tuned during NMS to optimize the balance between precision and recall. The model was trained for 50 epochs with a batch size of 64. The training was conducted on a computer with an Intel^®^ Xeon^®^ Gold 6130 processor (Intel, Santa Clara, CA, USA) and a NVIDIA Quadro^®^ RIXTM 4000 graphic processing unit (NVIDIA Corporate, Santa Clara, CA, USA).

Model performance was assessed at both the object detection and frame classification levels. Object-level metrics included recall, precision, mean average precision at IoU ≥ 0.50 (mAP50), and mean average precision across IoU thresholds from 0.50 to 0.95 (mAP50-95). Frame-level classification metrics included average precision, recall, and F1 score with corresponding 95% confidence intervals. A frame was considered a true positive if at least one correct polyp detection was present. The statistical analysis was performed using Sci-kit learn version 0.22.2 [25].

## 3. Results

On the test set, the model achieved a recall (equivalent to sensitivity) of 0.96, indicating it correctly identified 96% of true polyps, and a precision (equivalent to positive predictive value) of 0.95, indicating that 95% of the polyps detected by the model were true positives.

To assess localization performance at the object level, we applied an IoU threshold of 0.50, requiring that predicted bounding boxes overlap at least 50% with ground-truth annotation. Under this condition (mAP50), the model achieved a precision of 0.98. When evaluating performance across a range of more rigorous thresholds (from IoU 0.50 to 0.95, as in mAP50-95), the mean precision was 0.77.

To assess classification level performance, we computed precision and recall on a per-frame basis and then averaged these values across all frames. This approach yielded a mean recall of 0.75 (95% CI 0.73–0.77), a mean precision of 0.98 (95% CI 0.97–0.98), and a mean F1 score of 0.82 (95% CI 0.80–0.84).

Table 1 presents a detailed overview of the AI model’s performance on the test set.

**Figure 4:**
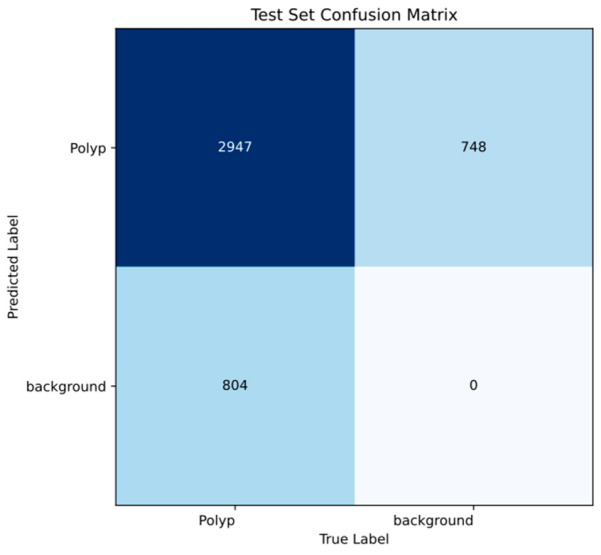
Confusion matrix of the automatic detection versus the expert’s classification in the testing dataset of the CNN model. Number of cases (relative frequency).

Figure 4 presents a frame-level confusion matrix, included to increase clinical interpretability. In this approach, a frame is considered a true positive if it contains at least one detected polyp. However, this simplification does not account for potential missed polyps within the same frame, as the model’s main evaluation was performed at the object (bounding box) level—vide supra. Therefore, this matrix should be interpreted with caution. Moreover, given the very limited number of background (non-polyp frames) in the test set, calculation of other clinically relevant metrics, such as specificity and negative predictive value, was not feasible.

## 4. Discussion

In this study, we developed and validated a CNN for the automatic detection and classification of lesions (polyps vs. other findings like myomas, cancer, and normal endometrium) from hysteroscopy images. This directly addresses the known challenges of subjectivity and inter-observer variability inherent in traditional visual interpretation of hysteroscopy. Our findings represent a significant step forward, particularly given the current scarcity of data on AI applications in hysteroscopic procedures.

The application of AI in hysteroscopy began nearly two decades ago. In 2006, one of the pioneering works in this field used texture image analysis algorithms to classify hysteroscopic images with the goal of early detection of gynecologic cancer. Using 418 images from 40 patients, they achieved a correct classification rate of 77% [26]. However, that novel research had notable drawbacks, including the pre-requisite for a physician to manually define regions of interest (ROIs) and the incomplete histopathological validation of some abnormal findings. Our study improves upon this by using a CNN that analyzes the entire image automatically and by ensuring every classification is validated by a histological ground truth.

After a notable gap in the literature until 2013, a Greek research group developed another neural network incorporating both texture and vasculature image features into their model. Using hysteroscopic images from 77 patients (10 of whom had confirmed endometrial carcinoma), they developed a tool capable of 91% classification accuracy [27]. While effective, their model relied on engineered features, whereas our deep learning approach allows the CNN to autonomously learn the most relevant patterns directly from the pixels, potentially capturing more complex features than those that were predefined.

More recently, research has accelerated and, in 2021, a Japanese study applied state-of-the-art deep learning architectures like Xception, MobileNetV2, and EfficientNetB0 on a dataset of 177 patients. Their results showed diagnostic accuracy between 80% and 90%, with sensitivity and specificity of 92% and 90%, respectively [28]. These findings highlight the potential of modern CNN architectures to improve clinical workflows.

That same year, a study from China developed a model to classify multiple hysteroscopic pathologies in a large cohort of 454 patients. Their work is relevant for using a large and histologically proven dataset. Their work is also interesting for directly comparing the CNN’s performance to that of gynecologists. They concluded that performance is superior with AI assistance, which is in line with recent evidence of AI use in clinical practice. The overall accuracy of their CNN was 81% [29]. One of the study’s limitation lies in using data from a single center, which may affect generalizability.

More recently, in a study submitted for publication in 2023, Li et al. explored the application of AI for fertility assessment in Asherman’s syndrome [30]. The group developed a CNN system trained on 4922 hysteroscopic images to predict conception outcomes and established a quantifiable visualization panel for intrauterine pathologies. While this work utilizes a different AI approach to the aforementioned studies and primarily focuses on prognosis in a specific subfertility condition rather than direct lesion classification of polyps, it underscores the expanding utility of AI within hysteroscopic imaging.

Our study builds upon these prior efforts by developing a CNN trained on multicentric, real-world data obtained from geographically and technologically distinct centers. The model is capable of simultaneously detecting and classifying polyps, offering both diagnostic and localization capabilities. The high performance observed at both object and frame levels suggest that the model is not only accurately identifying true polyps, but also correctly localizing them within hysteroscopic images. This localization is visually supported by bounding boxes, serving as a form of explainable AI. Such features are critical for clinical integration and trustworthiness, enhancing its technological readiness level and decision-support deployment.

We developed the CNN using a comprehensive, retrospectively collected dataset of hysteroscopies from three distinct high-volume centers from different continents, incorporating images from different hysteroscopy systems. This multicenter, multi-device approach is crucial for enhancing the potential generalizability of the CNN to diverse clinical settings.

We must also acknowledge some limitations, namely the retrospective nature of our data collection. Although the data came from multiple centers and current standards of practice were followed, prospective studies will be required. Although this study includes data from three centers over a one-year period, the number of included hysteroscopies is relatively small. This reflects the fact that not all procedures were recorded in video format, and among those that were, a subset was selected based on video quality and histological confirmation to support the development of a robust initial model. As this is a proof-of-concept study, our priority was to generate a reliable baseline system using the highest-quality data available.

As mentioned, our model allows detection and differentiation of polyps against other lesions, but in clinical practice it is essential that the CNN be able to detect and differentiate all types of lesions. In fact, this is a proof-of-concept model that focuses on differentiating polyps from other findings and future developments will enable multi-class classification of a broader range of pathologies. Additionally, because of the fact that the split is on the object level, there is a possibility of data leakage across splits, which could have had a modest impact on performance metrics. Ethical considerations, including data privacy and the prevention of algorithmic bias, were central to our study design, with full anonymization and adherence to GDPR. Continued vigilance in these areas will be critical as AI tools are translated into clinical practice. From an economic standpoint, the development and deployment of AI-based hysteroscopic tools will require initial costs, including software development, hardware integration, and regulatory approval. However, by potentially reducing diagnostic errors and standardizing care, such tools may offset costs through decreased need for repeated procedures or unnecessary biopsies. Further health-economic analyses are warranted to assess cost-effectiveness and inform clinical adoption.

One of the most promising applications of AI in hysteroscopy lies in its potential for real-time assistance during procedures. The integration of live image recognition tools into hysteroscopic systems would allow the AI to act as a “second observer” continuously analyzing the uterine cavity and highlighting areas of potential abnormality. This aligns with the increasing evidence of AI use in clinical practice, as stated in the work of the Chinese colleges that AI-supported diagnosis may surpass individual physician performance, particularly in pattern recognition tasks. As stated, our model, in its current state, was designed for polyp detection and classification; however, with further development, such systems can evolve for more sophisticated tools capable of lesion differentiation—distinguishing polyps from myomas, hyperplasia, and malignant lesions.

The future research of this group will focus on the development of models capable of multi-lesion detection and differentiation. Additional refinement efforts will include broadening the dataset to encompass a wider spectrum of endometrial pathologies, as well as images from diverse patient populations and more hysteroscopic systems to improve generalizability. Once more advanced models have been developed, it will be essential to conduct prospective validation in real-time clinical environments to confirm their performance, reliability, and clinical utility. This step is critical to ensure the model’s effectiveness in everyday practice and to support its integration into routine diagnostic workflows.

## 5. Conclusions

This proof-of-concept study presents a CNN-based model capable of automatically detecting and classifying polyps in hysteroscopic frames. Trained on multicenter, histologically validated data, the model offers a step forward in reducing diagnostic subjectivity. Future work will aim to expand lesion types, increase dataset diversity, and validate performance in real-time settings to enable effective integration into clinical practice.

## Figures and Tables

**Table 1:** Performance metrics of the AI model.

Evaluation Level	Metric	Value
Object level	Recall	0.96
	Precision	0.95
	Map50	0.98
	Map50-95	0.77
Frame level	Recall	0.75
	Precision	0.98
	Mean f1 score	0.82

## Data Availability

Non-identifiable data will be made available upon reasonable request.
